# High-cost users after sepsis: a population-based observational cohort study

**DOI:** 10.1186/s13054-024-05108-6

**Published:** 2024-10-21

**Authors:** Kali A. Barrett, Fatima Sheikh, Victoria Chechulina, Hannah Chung, Peter Dodek, Laura Rosella, Kednapa Thavorn, Damon C. Scales, Sepsis Canada

**Affiliations:** 1 Institute of Health Policy, Management and Evaluation, University of Toronto, Toronto, ON Canada; 2 Department of Medicine, Temerty Faculty of Medicine, University of Toronto, Toronto, ON Canada; 3 ICES, Toronto, ON Canada; 4 Toronto General Hospital Research Institute, University Health Network, Eaton Building, 10th Floor, 200 Elizabeth Street, Toronto, ON M5G 2C4 Canada; 5 Department of Health Research Methods, Evidence, and Impact, McMaster University, Hamilton, ON Canada; 6 Department of Epidemiology and Biostatistics, Western University, London, ON Canada; 7 Centre for Advancing Health Outcomes and Division of Critical Care Medicine, St. Paul’s Hospital and University of British Columbia, Vancouver, British Columbia Canada; 8 Division of Epidemiology, University of Toronto, Toronto, ON Canada; 9 Department of Laboratory Medicine and Pathobiology, Temerty Faculty of Medicine, University of Toronto, Toronto, ON Canada; 10 Institute for Better Health, Trillium Health Partners, Mississauga, ON Canada; 11 Ottawa Hospital Research Institute, Ottawa, ON Canada; 12 School of Epidemiology and Public Health, University of Ottawa, Ottawa, ON Canada; 13 Department of Critical Care, Sunnybrook Health Sciences Centre, North York, ON Canada; 14 Interdepartmental Division of Critical Care Medicine, University of Toronto, Toronto, ON Canada

**Keywords:** Sepsis, High-cost users, Healthcare utilization, Healthcare costs

## Abstract

**Background:**

High-cost users (HCU) represent important targets for health policy interventions. Sepsis is a life-threatening syndrome that is associated with high morbidity, mortality, and economic costs to the healthcare system. We sought to estimate the effect of sepsis on being a subsequent HCU.

**Methods:**

Using linked health-administrative databases, we conducted a population-based, propensity score-weighted cohort study of adults who survived a hospitalization in Ontario, Canada between January 2016 and December 2017. Sepsis was identified using a validated algorithm. The primary outcome was being a persistent HCU after hospital discharge (in the top 5% or 1% of total health care spending for 90 consecutive days), and the proportion of follow-up time since discharge as a HCU.

**Results:**

We identified 927,057 hospitalized individuals, of whom 79,065 had sepsis. Individuals who had sepsis were more likely to be a top 5% HCU for 90 consecutive days at any time after discharge compared to those without sepsis (OR 2.24; 95% confidence interval [CI] 2.04–2.46) and spent on average 42.3% of their follow up time as a top 5% HCU compared to 28.9% of time among those without sepsis (RR 1.46; 95% CI 1.45–1.48). Individuals with sepsis were more likely to be a top 1% HCU for 90 consecutive days compared to those without sepsis (10% versus 5.1%, OR 2.05 [95% CI 1.99–2.11]), and spent more time as a top 1% HCU (18.5% of time versus 10.8% of time, RR 1.68 [95% CI 1.65–1.70]).

**Conclusions:**

The sequelae of sepsis result in higher healthcare costs with important economic implications. After discharge, individuals who experienced sepsis are more likely to be a HCU and spend more time as a HCU compared to individuals who did not experience sepsis during hospitalization.

**Supplementary Information:**

The online version contains supplementary material available at 10.1186/s13054-024-05108-6.

## Background

Sepsis is a global health threat responsible for an estimated 20% of deaths worldwide^1^. This life-threatening health condition is also associated with economic costs in high-, middle-, and low-income countries and is associated with significant morbidity [1–6]. Sepsis survivors experience an increased risk of cardiovascular disease, long-term cognitive and functional impairment, worsening of pre-existing health chronic health conditions, and an increased risk of rehospitalization and death [2, 4, 6–8]. Sepsis and its long-term consequences are associated with significant costs to healthcare systems [9–11].

Individuals who have the highest health care needs in a population, often referred to as “High-Cost High-Need” or “High-Cost Users” (HCUs) represent a small percentage of the overall population, but account for a disproportionate amount of healthcare spending. Canadian [12], US [13], British [14], and Australian [15] studies have all shown that the top 5% of the population account for about 50% of total healthcare dollars spent [16]. These costs are highly skewed: a Canadian study reported that the top 1% of healthcare users accounted for 27.5% of total healthcare spending, as compared to the bottom 50% who incurred less than 4% of total healthcare costs [12]. Interventions aimed at preventing individuals from becoming a HCU by improving health and well-being could result in significant cost savings to the health system. Previous research has sought to understand the interplay between social determinants of health and becoming a HCU [17–19], but less is known about how specific health events create incident and persistent HCUs.

Although the long-term health consequences and costs after sepsis are well described [4, 6, 7], it is not known if individuals who survive a hospitalization with sepsis are more likely to become a HCU after discharge. The objective of this study was to determine if an episode of sepsis during a hospitalization increases an individual’s risk of becoming a HCU, compared to a hospitalization without sepsis.

## Methods

### Setting, population

The setting for this study is the province of Ontario (population 16 million) [12], which has a publicly funded, universal healthcare system. This population-based, propensity score-weighted cohort study was conducted using individual-level health administrative datasets for the province of Ontario, which contain records for all acute care hospitalizations in the province [13]. Individual records are linked using unique encoded identifiers and analyzed at ICES. Details regarding dataset validation and data access protocols are reported elsewhere [13]. ICES is an independent, non-profit research institute whose legal status under Ontario’s health information privacy law allows it to collect and analyze health care and demographic data, without consent, for health system evaluation and improvement. The use of the data in this project is authorized under Sect. 45 of Ontario’s Personal Health Information Protection Act (PHIPA) and does not require review by a Research Ethics Board. (Please see Supplementary Material 1: Appendix 1 for a description of ICES datasets used). The results of our study are reported according to the Reporting of studies Conducted using Observational Data (RECORD). [14]

### Cohort creation

We identified all adults aged ≥ 18 years who were eligible for publicly funded healthcare, who had no previous diagnosis of sepsis during a 2-year lookback period, and who survived an acute, non-obstetric hospitalization between January 1, 2016, and December 31, 2017. For individuals who had multiple hospitalizations during this accrual period, we selected the first. Hospitalization admission date was used as the index date, from which baseline covariates were collected and the look-back started. We considered the hospitalization to be the exposure, and we therefore did not include time during the hospitalization in the observed follow-up period. Follow-up time started the day after discharge from the hospital, and individuals were followed until death or administrative censoring on December 31, 2019, whichever was earliest.

### Exposure

Among our hospitalized cohort, we identified individuals exposed to sepsis using a previously described algorithm that uses ICD-10 codes for sepsis, organ dysfunction, and infection in the discharge abstract database [15]. (Please see Supplementary Material 1: Appendix 2 for details). Individuals who did not have sepsis identified during their index hospitalization were the unexposed comparator population.

### Covariates

We identified the following covariates at index date: age, sex, rurality, neighbourhood income quintile, area-level ecological markers of social determinants of health using the Ontario Marginalization index [16], and if the individual had immigrated to Canada prior to index date. We used a one-year lookback to identify markers of previous healthcare resource utilization including previous hospitalizations, emergency department visits, physician visits, healthcare costs, and whether individuals’ healthcare costs were in the top 10% of spending for each 30-day period, (see methods for healthcare costs below). We used a two-year lookback to identify whether the individual had resided in a long-term care home, and to identify the presence of medical comorbidities that are known to predispose to sepsis and healthcare resource utilization (cancer, congestive heart failure, chronic obstructive pulmonary disease, chronic kidney disease or chronic dialysis, and diabetes) [17]. We used the Johns Hopkins ACG® System Version 10, which is a multi-morbidity framework that groups individuals based on expected future healthcare resource utilization, to identify frailty, resource utilization bands, and aggregated diagnosis groups (ADGs). [18–20]

We identified the following covariates for the index hospitalization: hospital size, type of hospitalization based on case mix groups (medical, surgical, other), Charlson score, hospital length of stay, admission to an intensive care unit (ICU), ICU length of stay, receipt of mechanical ventilation (non-invasive and invasive), receipt of extracorporeal life support (ECLS), healthcare costs for the index hospitalization, and discharge location. See Supplementary Material 1: Appendix 3 for a list of sources for all covariates.

### Propensity score weighting

We calculated the propensity score by regressing sepsis exposure on 17 baseline covariates selected based on previous research and our clinical understanding of the risk factors for sepsis and for future healthcare resource utilization (see Supplementary Material 1: Appendix 4) [2, 21–25]. Hospital and ICU length of stay, and the need for mechanical ventilation were not included as covariates in the propensity score model, as we viewed these as mediators rather than confounding factors. Adding mediators to IPTW alongside confounders can lead to overadjustment bias, induce collider bias, and distort the effect estimation. We did not believe ICU admission was a mediator and therefore we did include this covariate in the propensity score model. To adjust for confounding, we calculated inverse probability of treatment weights (IPTW) for each individual using their propensity score and exposure status. [26, 27] Compared to propensity score matching, the use of IPTW to control for confounding has several advantages including inclusion of all individuals in the dataset, ability to achieve balance across a range of covariates, and improved overall precision [26]. We assessed the balance of covariates between the two groups before and after weighting by comparing the weighted standardized difference (WSD), using an acceptable threshold of < 10%.

### Outcomes

We estimated total healthcare costs for each individual in our cohort in 30-day intervals from hospital discharge to the end of follow-up using a hybrid costing methodology developed for administrative databases. [28, 29] The algorithm identifies healthcare encounters such as physician visits, diagnostic tests, same-day surgery, hospitalizations, emergency department visits, rehabilitation, homecare, prescription drugs, complex continuing care, and long-term care. Costs were calculated for each encounter using the unit cost or by multiplying the resource intensity weights specific to each database by the average provincial cost per weighted case. Costs were standardized to 2020 Canadian dollar values. [29]

The primary outcome was HCU status after hospitalization. HCU status was identified by comparing individual healthcare costs per 30-day period to the pre-identified top 5% cost threshold specific to that same 30-day calendar period (further details available in Supplementary Material 1: Appendix 5 and 6). Individuals whose 30-day costs were at or above the thresholds were a HCU compared to total population health spending for that time-period. We report the primary outcome as being a persistent HCU (the top 5%) for any period of 90 consecutive days (3 consecutive 30-day periods) after discharge from hospital. We also report the proportion of individual subjects’ follow-up time spent as a HCU since hospital discharge, which was calculated using individual duration of follow-up time as the denominator. We repeated all analyses defining HCU at the top 1% threshold, which is a more specific definition of HCUs, and places individuals among the very top of healthcare spending.

### Subgroup analyses

Previous studies exploring trajectories of HCU in Ontario have demonstrated that almost half of individuals in the top 10% of healthcare spending continue to be in the top 10% in subsequent years. [25] We therefore expected that many individuals in our cohorts would be pre-existing HCUs. To better understand the impact of sepsis among those who were not previously a HCU, we conducted additional analysis stratifying individuals based on whether they had ever been in the top 10% of healthcare spending for 30-days in the year prior to hospitalization. We recalculated the propensity score for sepsis within each strata (previous top 10%, not previously in top 10%) and repeated IPTW weighting within each strata. Baseline characteristics of these two stratified cohorts before and after weighting are presented in Supplementary Material 1: Appendix 7.

### Statistical analyses

We used a weighted logistic regression with robust standard error to estimate the odds ratio (OR) of becoming a persistent HCU (any period of 90 consecutive days as a HCU) comparing individuals who were hospitalized with sepsis to those who did not have sepsis. We used weighted Poisson regression modeling with robust standard error to estimate the relative rate of being a HCU at any point during follow-up time. All statistical analyses were done using SAS® software, Version 9.4 (SAS Institute Inc., Cary, NC, USA).

## Results

**Fig. 1:**
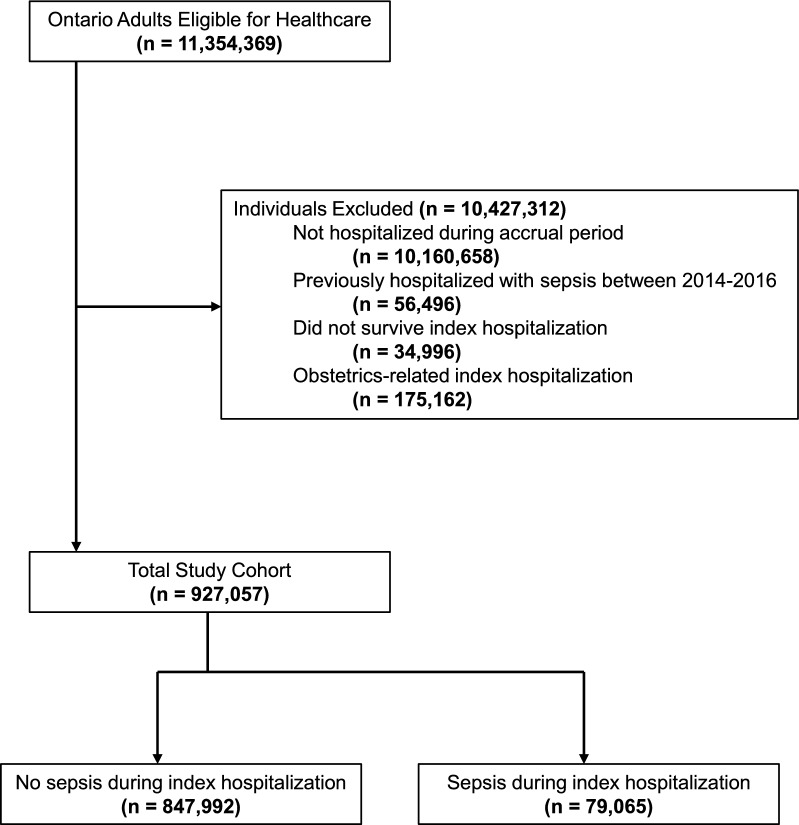
Cohort Creation Flow Diagram

There were 927,057 adults who survived an admission to an Ontario hospital for a non-obstetric reason in 2016 and 2017, among whom we identified an episode of sepsis in 79,065 (see Table 1 and Fig. 1). Individuals experiencing sepsis were older (mean age 72.8 [SD 16.3] versus 60.2 [SD 19.1]) years old), more likely to be frail (22.3% versus 8.2%), and more likely to have had a hospitalization in the year prior (8.8% versus 6.3%). Almost all (95.0% sepsis versus 92.3% non-sepsis) individuals in the cohort had at least one 30-day period during the 2 years prior to the hospitalization in which their 30-day total healthcare cost was in the top 10%. After weighting, baseline characteristics were balanced between the two cohorts for all covariates, except for frailty (12.9% sepsis versus 9.24% non-sepsis [WSD 0.12]), and among those in the highest band of previous resource utilization (27.9% sepsis versus 23.3% non-sepsis [WSD 0.11]).

**Table 1:** Baseline characteristics of individuals with and without sepsis during hospitalization

Variable	Prior to weighting			After weighting		
	Sepsis N = 79, 065	Non-Sepsis N = 847, 992	Standardized Difference	Sepsis N = 888,513	Non-Sepsis N = 928,323	Weighted Standardized Difference
**Age,** mean ± SD median (IQR)	72.77 ± 16.25 76 (64–85)	60.19 ± 19.06 63 (47–75)	0.71 0.72	62.14	61.29	0.044
**Female,** n (%)	44,556 (56.4%)	457,932 (54.0%)	0.05	52.01%	54.19%	0.044
**Immigration Status**, n (%)						
Immigrated prior to index date	6,496 (8.2%)	104,705 (12.3%)	0.14	11.13%	11.98%	0.026
Non-immigrant	72,569 (91.8%)	743,287 (87.7%)	0.14	88.87%	88.02%	0.026
Recent immigrant (≤ 5 years)	387 (0.5%)	10,701 (1.3%)	0.08	0.83%	1.20%	0.037
Long-term immigrant (> 5 years)	6,109 (7.7%)	94,004 (11.1%)	0.12	10.30%	10.78%	0.016
Rural, n (%)	7,834 (9.9%)	82,088 (9.7%)	0.01	9.87%	9.70%	0.006
**Neighborhood Income Quintile, n (%)**						
1 (lowest)	20,798 (26.3%)	187,527 (22.1%)	0.1	24.10%	22.50%	0.038
2	17,439 (22.1%)	177,345 (20.9%)	0.03	21.49%	21.00%	0.012
3	14,993 (19.0%)	167,404 (19.7%)	0.02	19.45%	19.66%	0.005
4	13,262 (16.8%)	157,678 (18.6%)	0.05	17.92%	18.42%	0.013
5 (highest)	12,200 (15.4%)	155,285 (18.3%)	0.08	16.66%	18.09%	0.038
Missing information	373 (0.5%)	2,753 (0.3%)	0.02	0.37%	0.35%	0.004
*Ontario marginalization index dimensions*						
**Household and Dwellings, n (%)**						
1 (least deprived)	10,170 (12.9%)	143,863 (17.0%)	0.12	15.39%	16.62%	0.034
2	12,753 (16.1%)	153,245 (18.1%)	0.05	17.34%	17.89%	0.014
3	14,565 (18.4%)	161,287 (19.0%)	0.02	18.44%	18.96%	0.013
4	16,912 (21.4%)	169,093 (19.9%)	0.04	20.35%	20.07%	0.007
5 (most deprived)	23,434 (29.6%)	210,407 (24.8%)	0.11	27.10%	25.22%	0.043
Missing information	1,231 (1.6%)	10,097 (1.2%)	0.03	1.38%	1.23%	0.013
**Material Resources, n (%)**						
1 (least deprived)	13,434 (17.0%)	166,239 (19.6%)	0.07	18.33%	19.37%	0.026
2	14,457 (18.3%)	165,606 (19.5%)	0.03	18.77%	19.40%	0.016
3	14,829 (18.8%)	162,359 (19.1%)	0.01	19.08%	19.12%	0.001
4	16,126 (20.4%)	166,961 (19.7%)	0.02	20.14%	19.75%	0.010
5 (most deprived)	18,988 (24.0%)	176,730 (20.8%)	0.08	22.31%	21.13%	0.029
Missing information	1,231 (1.6%)	10,097 (1.2%)	0.03	1.38%	1.23%	0.013
**Age and Labour Force, n (%)**						
1 (least deprived)	10,888 (13.8%)	162,257 (19.1%)	0.15	17.68%	18.68%	0.026
2	12,413 (15.7%)	153,407 (18.1%)	0.06	17.70%	17.90%	0.005
3	13,190 (16.7%)	149,258 (17.6%)	0.02	17.62%	17.51%	0.003
4	15,324 (19.4%)	157,636 (18.6%)	0.02	18.83%	18.63%	0.005
5 (most deprived)	26,019 (32.9%)	215,337 (25.4%)	0.17	26.80%	26.06%	0.017
Missing information	1,231 (1.6%)	10,097 (1.2%)	0.03	1.38%	1.23%	0.013
**Racialized and Newcomer Populations, n (%)**						
1 (least deprived)	18,166 (23.0%)	177,230 (20.9%)	0.05	21.28%	21.07%	0.005
2	16,646 (21.1%)	164,547 (19.4%)	0.04	20.20%	19.49%	0.018
3	14,442 (18.3%)	155,651 (18.4%)	0	18.10%	18.36%	0.007
4	13,544 (17.1%)	159,303 (18.8%)	0.04	17.79%	18.69%	0.023
5 (most deprived)	15,036 (19.0%)	181,164 (21.4%)	0.06	21.26%	21.16%	0.002
Missing information	1,231 (1.6%)	10,097 (1.2%)	0.03	1.38%	1.23%	0.013
**Resource Utilization Bands, n (%)**						
Band 0 (non-users)	926 (1.2%)	9,448 (1.1%)	0.01	1.44%	1.09%	0.031
Band 1	457 (0.6%)	5,536 (0.7%)	0.01	0.92%	0.63%	0.033
Band 2	2,314 (2.9%)	34,099 (4.0%)	0.06	4.14%	3.90%	0.012
Band 3	25,731 (32.5%)	327,205 (38.6%)	0.13	36.94%	38.05%	0.023
Band 4	22,316 (28.2%)	283,066 (33.4%)	0.11	28.64%	33.09%	0.096
Band 5 (very high users)	27,321 (34.6%)	188,638 (22.2%)	0.28	27.92%	23.25%	0.107
Frailty indicator (ACGs), n (%)	17,671 (22.3%)	69,630 (8.2%)	0.4	12.92%	9.24%	0.118
Number of ACGs, mean ± SD, median (IQR)	8.67 ± 4.04 9 (6–11)	8.00 ± 3.79 8 (5–11)	0.17 0.17	8.33	8.06	0.067
Number of ACGs (0), n (%)	928 (1.2%)	9,470 (1.1%)	0.01	1.44%	1.09%	0.031
Number of ACGs (1–9), n (%)	45,302 (57.3%)	554,494 (65.4%)	0.17	60.56%	64.72%	0.086
Number of ACGs (10–19), n (%)	32,574 (41.2%)	282,299 (33.3%)	0.16	37.73%	33.94%	0.079
Number of ACGs (20–29), n (%)	261 (0.3%)	1,729 (0.2%)	0.02	0.27%	0.26%	0.003
Prior Cancer, n (%),	18,260 (23.1%)	153,062 (18.0%)	0.13	19.40%	18.49%	0.023
Prior Congestive Heart Failure, n (%)	14,711 (18.6%)	74,383 (8.8%)	0.29	10.78%	9.67%	0.037
Prior chronic kidney disease or chronic dialysis, n (%)	8,571 (10.8%)	47,604 (5.6%)	0.19	7.22%	6.09%	0.045
Prior chronic obstructive pulmonary disease, n (%)	26,963 (34.1%)	159,031 (18.8%)	0.35	22.07%	20.12%	0.048
Prior diabetes, n (%)	28,374 (35.9%)	203,236 (24.0%)	0.26	26.82%	25.04%	0.041
**Healthcare Use 1-year Prior Index**						
ED visits, mean ± SD, median (IQR)	1.57 ± 2.51 1 (0–2)	1.26 ± 2.60 1 (0–2)	0.12 0.25	1.54	1.41	0.027
Receipt of home care, n (%)	27,260 (34.5%)	132,322 (15.6%)	0.45	19.75%	17.32%	0.063
Hospitalization, n (%)	6,983 (8.8%)	53,320 (6.3%)	0.1	7.51%	6.55%	0.038
No. of hospitalization episodes, mean ± SD, median (IQR)	0.13 ± 0.49 0 (0–0)	0.09 ± 0.44 0 (0–0)	0.08 0.1	0.11	0.1	0.026
No. of physician visits, mean ± SD, median (IQR)	11.52 ± 10.61 9 (5–15)	11.36 ± 9.54 9 (5–15)	0.02 0.02	12.14	11.39	0.064
LTC resident in 2-year lookback, n (%)	6,443 (8.1%)	15,227 (1.8%)	0.3	2.86%	2.40%	0.029
Previous top 10%, n (%)	75,105 (95.0%)	782,399 (92.3%)	0.11	93.14%	92.51%	0.024

*SD* Standard Deviation, *IQR* Interquartile Range, *ACG* Adjusted Clinical Groups, Frailty, and Resource utilization bands from the Johns Hopkins ACG® System Version 10*, OnMARG* Ontario Marginalization Index, *LTC* Long-term Care Resident

About half (51.3%) of hospitalizations with sepsis were to large hospitals with over 100 beds (Table 2). Many sepsis hospitalizations (60.6%) were classified as medical, while the non-sepsis hospitalizations were equally distributed between medical, surgical, and other categories. Even after propensity score weighting, individuals with sepsis had longer mean hospital length of stay (12.8 versus 5.05 days [WSD 0.381]), were more frequently admitted to the ICU (17.6% versus 15.0% [WSD 0.069]), had longer mean ICU length of stay (9.95 versus 3.15 days [WSD 0.510]) and were more likely to receive mechanical ventilation (9.41% versus 4.26% [WSD 0.205]). The average cost of the index hospitalization in the weighted cohort was also higher among those with sepsis ($17,395 versus $7,925 [WSD 0.343]).

**Table 2:** Characteristics of index hospitalization among individuals with and without sepsis

Variable	Prior to weighting			After weighting		
	Sepsis N = 79, 065	Non-Sepsis N = 847, 992	Standardized Difference	Sepsis N = 888,513	Non-Sepsis N = 928,323	Weighted Standardized Difference
**Hospital Size, n (%)**						
Teaching	20,511 (25.9%)	279,789 (33.0%)	0.16	27.41%	32.85%	0.119
Community ≥ 100 beds	40,551 (51.3%)	424,804 (50.1%)	0.02	51.30%	50.06%	0.025
Community < 100 beds	16,758 (21.2%)	128,145 (15.1%)	0.16	19.77%	15.28%	0.118
Missing information	1,245 (1.6%)	15,254 (1.8%)	0.02	1.51%	1.81%	0.023
**Type of hospitalization, n (%)**						
Medical (including mental health)	47,903 (60.6%)	287,902 (34.0%)	0.55	60.51%	34.61%	0.537
Surgical	8,159 (10.3%)	291,135 (34.3%)	0.6	10.76%	33.89%	0.578
Not categorized	23,003 (29.1%)	268,955 (31.7%)	0.06	28.74%	31.51%	0.060
Charlson score of index hospitalization, mean ± SD, median (IQR)	1.51 ± 1.68 1 (0–2)	0.89 ± 1.46 0 (0–1)	0.39 0.52	1.24	0.92	0.207
Length of Stay (days), mean ± SD, median (IQR)	14.79 ± 28.89 7 (4–15)	4.88 ± 9.91 3 (1–5)	0.46 0.96	12.82	5.05	0.381
ICU Admission during index admission, n (%)	16,750 (21.2%)	121,991 (14.4%)	0.18	17.57%	15.03%	0.069
ICU length of stay (days), mean ± SD, median (IQR)	8.84 ± 17.42 5 (2–9)	3.14 ± 4.96 2 (1–4)	0.45 0.81	9.95	3.15	0.510
Mechanical ventilation, n (%)	8,301 (10.5%)	35,006 (4.1%)	0.25	9.41%	4.26%	0.205
ECLS during index admission, n (%)	151 (0.2%)	354 (0.0%)	0.04	0.21%	0.04%	0.046
**Discharge location, n (%)**						
Transferred to acute inpatient facility	955 (1.2%)	6,730 (0.8%)	0.04	1.19%	0.81%	0.038
Transferred to long-term care, continuing care, or supportive housing	17,867 (22.6%)	64,046 (7.6%)	0.43	14.51%	8.37%	0.194
Discharged home	59,559 (75.3%)	767,584 (90.5%)	0.41	82.80%	89.72%	0.202
Discharged other [e.g., AMA, on pass]	684 (0.9%)	9,632 (1.1%)	0.03	1.51%	1.11%	0.035
Cost of index hospitalization, Mean ± SD, Median (IQR)	$18,434.82 ± $37,142.81 $8,713 ($5,486—$16,687)	$7,788.10 ± $10,962.88 $5,212 ($3,269—$8,372)	0.39 0.78	$17,394.6	$7924.88	0.343

*SD* Standard Deviation, *IQR* Interquartile Range, *ECLS* Extracorporeal life support

aThis includes invasive or non-invasive mechanical ventilation

Compared to individuals who did not have sepsis during their index hospitalization, those who had sepsis were more likely to have healthcare spending that places them in the top 5% (OR 1.73 [95% CI 1.70–1.77]) or top 1% (OR 2.05 [95% CI 1.99–2.11]) of healthcare spending for 90 consecutive days at any time during follow up (Table 3).

**Table 3:** High-cost user status post-discharge for those with and without sepsis during hospitalization

Variable	Unweighted			Weighted		
	Sepsis	Non-Sepsis	Measure of Association	Sepsis	Non-Sepsis	Measure of Association
*Total cohort*						
	N = 79, 065	N = 847, 992		N = 888,513	N = 928,323	
Death during follow-up, n (%)	30,158 (38.1%)	127,925 (15.1%)		25.60%	16.41%	
High-Cost User (Top 5%) for any 90-day period after discharge from hospital, n (%)	35,419 (44.8%)	201,533 (23.8%)	OR 2.60 (95% CI 2.56, 2.64)	36.51%	24.91%	OR 1.73 (95% CI 1.70, 1.77)
High-Cost User (Top 1%) for any 90-day period after discharge from hospital, n (%)	10,082 (12.8%)	40,282 (4.8%)	OR 2.93 (95% CI 2.86, 3.00)	9.96%	5.13%	OR 2.05 (95% CI 1.99, 2.11)
Proportion of follow-up time as a High-Cost User (Top 5%), Mean ± SD, Median (IQR)	55.04% ± 39.22 52% (15–100)	27.33% ± 33.18 12% (3–39)	RR 2.01 (95% CI 2.01, 2.02)	42.27%	28.89%	RR 1.46 (95% CI 1.45, 1.48)
Proportion of follow-up time as a High-Cost User (Top 1%), Mean ± SD, Median (IQR)	24.48 ± 33.11 8 (1–33)	10.09 ± 22.33 2 (0–7)	RR 2.43 (95% CI 2.42, 2.43)	18.15%	10.83%	RR 1.68 (95% CI 1.65, 1.70)
Total length of follow-up (days), Mean ± SD, Median (IQR)	839.51 ± 437.37 934 (531 – 1,189)	1,016.06 ± 342.51 1,063 (854 – 1,281)		930.17	1006.42	
*Previously in top 10%*						
	N = 75, 105	N = 782, 399		N = 821,753	N = 858,653	
High-Cost User (Top 5%) for any 90-day period after discharge from hospital, n (%)	34,275 (45.6%)	194,921 (24.9%)	OR 2.53 (95% CI 2.49, 2.57)	37.62%	26.07%	OR 1.71 (95% CI 1.68, 1.74)
High-Cost User (Top 1%) for any 90-day period after discharge from hospital, n (%)	9,673 (12.9%)	38,716 (4.9%)	OR 2.84 (95% CI 2.77, 2.91)	10.14%	5.33%	OR 2.00 (95% CI 1.94, 2.07)
Proportion of follow-up time as a High-Cost User (Top 5%), Mean ± SD, Median (IQR)	56.40% ± 38.88 56% (17–100)	28.78% ± 33.62 13% (4–43)	RR 1.96 (95% CI 1.96, 1.96)	43.94%	30.37%	RR 1.45 (95% CI 1.44, 1.46)
Proportion of follow-up time as a High-Cost User (Top 1%), Mean ± SD, Median (IQR)	25.04 ± 33.31 9 (2–35)	10.62 ± 22.84 2 (0–8)	RR 2.36 (95% CI 2.35, 2.36)	18.8%	11.38%	RR 1.65 (95% CI 1.63, 1.68)
*Not previously in top 10%*						
	N = 3, 960	N = 65, 593		N = 66,961	N = 69,578	
High-Cost User (Top 5%) for any 90-day period after discharge from hospital, n (%) l	1,144 (28.9%)	6,612 (10.1%)	OR 3.62 (95% CI 3.37, 3.90)	20.89%	10.54%	OR 2.24 (95% CI 2.04, 2.46)
High-Cost User (Top 1%) for any 90-day period after discharge from hospital, n (%)	409 (10.3%)	1,566 (2.4%)	OR 4.71 (95% CI 4.20, 5.28)	7.13%	2.50%	OR 2.99 (95% CI 2.60, 3.45)
Proportion of follow-up time as a High-Cost User (Top 5%), Mean ± SD, Median (IQR)	29.31% ± 36.79 10% (3–46)	10.05% ± 20.71 3% (0–8)	RR 2.92 (95% CI 2.90, 2.94)	19.39%	10.55%	RR 1.84 (95% CI 1.74, 1.94)
Proportion of follow-up time as a High-Cost User (Top 1%), Mean ± SD, Median (IQR)	13.93 ± 26.96 3 (0–11)	3.79 ± 13.48 0 (0–2)	RR 3.68 (95% CI 3.64, 3.71)	9.13%	4.01%	RR 2.28 (95% CI 2.10, 2.48)

*OR* Odds Ratio, *RR* Relative Rate, *SD* Standard Deviation, *IQR* Interquartile Range

Individuals who had sepsis during their hospitalization spent on average 42.3% of their follow up time as a top 5% HCU, compared to 28.9% among those without sepsis (RR 1.46 [95% CI 1.45–1.48]). They spent on average 18.2% of their follow-up time as a top 1% of HCU, compared to 10.8% among those without sepsis (RR 1.68 [95% CI 1.65–1.70]).

In the stratified analysis including only those who were not previously in the top 10% of health care costs, individuals with sepsis were a top 5% HCU for 19.4% of their observation time compared to 10.6% among those who did not have sepsis (RR 1.84 [95% CI 1.74–1.94]). The odds of being a top 1% HCU for 90 days after discharge was highest among this group (OR 2.99 [95% CI 2.60, 3.45]).

## Discussion

This study demonstrates that individuals who experience sepsis during a hospitalization are more likely to be a top 5% or top 1% high-cost user for any consecutive 90-day period after discharge and spend more time after discharge as a HCU compared to individuals hospitalized without sepsis. The odds of becoming a HCU after sepsis are highest among those who did not previously have high health-care costs, suggesting that sepsis can significantly change the trajectory of an individual’s healthcare utilization. We also demonstrate that hospitalizations with sepsis are more resource intensive compared to those without sepsis: they cost more, have higher rates of mechanical ventilation, and have longer hospital and ICU lengths of stay.

These results are concordant with previous studies that have identified increased healthcare costs, morbidity, and mortality after a hospitalization with sepsis [9–11]. Recent Ontario data showed that the annual mean healthcare costs for a cohort of individuals who survived a hospitalization with sepsis were significantly higher than a non-sepsis matched cohort, and that these costs remained high, exceeding $20,000 per year, at five years after discharge [2]. Our study adds to these data by demonstrating that these individual costs are among the highest when compared to total population-level health system spending.

Healthcare spending among HCU reflects increased use of inpatient services. A 2016 study examined the types of healthcare costs among top 5% and 1% high-cost users in Ontario. They found that costs related to inpatient health care (acute care, continuing care, or other hospital services) were the largest drivers of healthcare spending among HCU [30]. Among the top 1%, acute hospital costs represented over 30% of total costs, and among the top 1% who did not experience an acute care admission, continuing care represented about half of total costs [30]. Previous studies have demonstrated that readmission rates at 7-, 30-, and 90-days are significantly higher among individuals who experience sepsis during hospitalization compared to those who do not [8, 11, 31]. Recent evidence demonstrates that this increased risk of hospitalization persists for up to 5 years following sepsis (5-year hazard ratio 1.41 [95% CI 1.40–1.43] among those with sepsis, and 1.53 [95% CI 11.50–1.55] among those with severe sepsis) [2]. Sepsis survivors are known to be at a higher risk of death after discharge, and healthcare costs are known to increase before death [32]. It is likely that the costs incurred from hospital-readmission and end-of-life care are contributing to the increased costs among sepsis survivors in our study.

The accurate identification of exposure status can be challenging when using health administrative databases, and this represents a potential limitation in our study. We identified exposure to sepsis via an algorithm that uses ICD-10 codes for infection and organ dysfunction. This algorithm has been validated in ICU and non-ICU populations, with acceptable specificity (> 85%), but with much lower sensitivity (ranging from 25% to 71.9%) [15]. Milder cases of sepsis are known to be under-coded in health administrative databases [15]. This has potential implications for our results. If individuals with milder cases of sepsis experience an increase in healthcare costs after discharge, than this misclassification will bias our results towards the null. However, if milder cases do not experience increased healthcare costs after discharge, then the magnitude of effect due to sepsis that we identified may be inflated. We hope that the new SEPSIS-3 definition and a transition to ICD-11 coding in administrative records will increase the accuracy of sepsis coding and identification in health records.

Previous studies have sought to identify predictors of becoming a HCU. Social determinants of health are strong predictors of being a HCU. [21, 22, 33] Living in a lower income or more marginalized community is associated with increased risk of becoming a HCU [22–24]. Food insecurity has been noted as one of the strongest predictors of being a HCU [24, 34]. The presence of multiple chronic medical conditions is understandably associated with being a HCU [25]. Older age and female sex have also been identified as risk factors [21]. A key strength of this study is that we accounted for these known predictors of future HCU status [24, 35]. We adjusted for common pre-existing comorbidities and neighbourhood level markers of marginalization and income in our propensity score model. We recognise that neighbourhood level markers of social determinants of health need to be handled cautiously, as they do not always perfectly align with individual level income or demographic data [36]. However, these covariates were well balanced after weighting, and our results demonstrate that after using best available methods to control for these known predictors of future healthcare use, sepsis remains a strong independent risk factor for becoming a future HCU.

## Conclusion

This study illustrates the impact of sepsis using a well-known marker of healthcare utilization (HCU) that is of interest to health economists and health-system decision-makers. Our results suggest that sepsis can significantly change the trajectory of healthcare utilization among those who were not previously a HCU. These results have important public health and health system implications, and they highlight the economic consequences of sepsis. Programs and policies that aim to prevent sepsis, to reduce the clinical burden of sepsis, and to improve outcomes after sepsis could have significant economic benefit by averting new HCUs. Future research is needed to better predict who is at greatest risk of becoming a HCU after sepsis, and how important social determinants of health modify this risk.

## Supplementary Information

Supplementary Material 1: Appendix 1–8

## Data Availability

The dataset from this study is held securely in coded form at ICES. While legal data sharing agreements between ICES and data providers (e.g., healthcare organizations and government) prohibit ICES from making the dataset publicly available, access may be granted to those who meet pre-specified criteria for confidential access, available at www.ices.on.ca/DAS (email: das@ices.on.ca). The full dataset creation plan and underlying analytic code are available from the authors upon request, understanding that the computer programs may rely upon coding templates or macros that are unique to ICES and are therefore either inaccessible or may require modification.

## Abbreviations

HCU: High-cost user
PHIPA: Personal Health Information Protection Act
RECORD: Reporting of studies conducted using observational data
ADG: Aggregated diagnosis groups
ICU: Intensive care unit
ECLS: Extracorporeal life support
IPTW: Inverse probability of treatment weights
WSD: Weighted standardized difference
