# Supplementary material for: High-cost users after sepsis: a population-based observational cohort study
Source: Crit Care. 2024 Oct 21;28:338. doi: 10.1186/s13054-024-05108-6 (PMC11492703; doi:10.1186/s13054-024-05108-6)
Supplement: Supplementary file 1 — Supplementary Material 1: Appendix 1–8 [file 13054_2024_5108_MOESM1_ESM.docx]

**Supplementary Appendix**

High-cost users after sepsis: A population-based observational cohort study.

**Authors:**

Kali A Barrett, MD MSc^1-4^ (kali.barrett@uhn.ca), Fatima Sheikh, MSc^5^ (sheikf9@mcmaster.ca), Victoria Chechulina^6^ ([vchechul@uwo.ca](mailto:vchechu4@gmail.com)), Hannah Chung, MPH^3^ ([hannah.chung@mail.utoronto.ca](mailto:hannah.chung@mail.utoronto.ca)), Peter Dodek, MD MHSc^7^ (peter.dodek@ubc.ca), Laura Rosella, MHSc PhD^3,8-10^ ([laura.rosella@utoronto.ca](mailto:laura.rosella@utoronto.ca)) Kednapa Thavorn, MPharm PhD^3,11,12^([kthavorn@ohri.ca](mailto:kthavorn@ohri.ca)), Damon C. Scales, MD PhD^1,3,13,14^ (damon.scales@sunnybrook.ca); on behalf of Sepsis Canada

**Affiliations:**

1. Institute of Health Policy, Management and Evaluation, University of Toronto, Ontario, Canada
2. Department of Medicine, Temerty Faculty of Medicine, University of Toronto, Ontario, Canada
3. ICES, Toronto, Ontario, Canada
4. Toronto General Hospital Research Institute, University Health Network, Toronto, Ontario, Canada
5. Department of Health Research Methods, Evidence, and Impact, McMaster University, Ontario, Canada
6. Department of Epidemiology and Biostatistics, Western University, London, Ontario, Canada
7. Centre for Advancing Health Outcomes and Division of Critical Care Medicine, St. Paul's Hospital and University of British Columbia, British Columbia, Canada
8. Division of Epidemiology, University of Toronto, Ontario, Canada
9. Department of Laboratory Medicine and Pathobiology, Temerty Faculty of Medicine, University of Toronto, Ontario, Canada
10. Institute for Better Health, Trillium Health Partners, Ontario, Canada
11. Ottawa Hospital Research Institute, Ontario, Canada
12. School of Epidemiology and Public Health, University of Ottawa, Ontario, Canada
13. Department of Critical Care, Sunnybrook Health Sciences Centre, Ontario Canada
14. Interdepartmental Division of Critical Care Medicine, University of Toronto, Ontario, Canada

**Corresponding author:**

Dr Kali Barrett

University Health Network 
Toronto General Hospital 
Eaton Building, 10^th^ Floor 
200 Elizabeth Street

Toronto, ON, Canada

M5G 2C4 
[Kali.barrett@uhn.ca](mailto:Kali.barrett@uhn.ca)

416-340-4800

**Contents**

Supplementary Appendix 1: List of ICES Datasets Used

Supplementary Appendix 2: ICD-10-CA Codes Used to Define Sepsis Cases

# Supplementary Appendix 3: List of Covariates and Datasets

Supplementary Appendix 4: Covariates Included in Propensity Score Model

Supplementary Appendix 5: Classification of High-Cost User

Supplementary Appendix 6: Monthly Top 5% and Top 1% High-Cost User Thresholds for 2014-2019 in 2020 Canadian dollars.

Supplementary Appendix 7: Baseline characteristics stratified by previous healthcare costs (top 10%) pre and post propensity score weighting

Supplementary Appendix 8: Record Checklist

**Supplementary Appendix 1: List of ICES Datasets Used**

| **Dataset** | **Description** |
| --- | --- |
| ADP | Assistive Devices Program |
| AVGPRICE | Average Price |
| CAPE | Client Agency Program Enrolment |
| CCIS | Critical Care Information System |
| CCRS | Continuing Care Reporting System |
| CENSUS | Ontario Census Area Profiles |
| CHF | Congestive Heart Failure ICES Cohort |
| CIHI DAD | Canadian Institute for Health Research Discharge Abstract Database |
| CONTACT | Yearly Health Services Contact |
| COPD | Chronic Obstructive Pulmonary Disease ICES Cohort |
| CORR | Candian Organ Replacement Registry |
| GAPP | GAPP Decision Support Systems (Physician Payments) |
| HCD | Home Care Database |
| INST | Information about Ontario health care institutions funded by the Ministry of Health and Long-Term Care (MOHLTC) |
| IRCC | Immigration, Refugees and Citizenship Canada’s Permanent Resident Database |
| LHIN | Local Heatlh Integration Network |
| NACRS | National Ambulatory Care Reporting System |
| NDFP | New Drug Funding Program |
| NRS | National Rehabilitation Reporting System |
| OCCI | Ontario Case Costing Initiative |
| OCR | Ontario Cancer Registry |
| ODB | Ontario Drug Benefit Claims |
| ODD | Ontario Diabetes Dataset |
| OHIP | Ontario Health Insurance Plan Claims Database |
| ONMARG | Ontario Marginalization Index |
| ORRS | Ontario Renal Reporting System |
| PCCF | Postal Code Conversion File |
| RAIHC | inter Resident Assessment Instrument (RAI) - Home Care Source (HSSO) |
| REF | Reference Files (Look-up Tables) |
| RPDB | Registered Persons Database |
| SDS | Same Day Surgery Database (Annual) |

**Supplementary Appendix 2: ICD-10-CA Codes Used to Define Sepsis Cases**

| **JOLLEY ET AL. ALGORITHM** | |
| --- | --- |
| **Sepsis** | **Severe Sepsis (Including Septic Shock)** |
| A03.9, A02.1, A20.7, A21.7, A22.7, A23.9, A24.1, A26.7, A28.0, A28.2, A32.7, A39.2, A39.3, A39.4, A40, A40.0, A40.1, A40.2, A40.3, A40.8, A40.9, A41, A41.0, A41.1, A41.2, A41.3, A41.4, A41.5, A41.50, A41.51, A41.52, A41.58, A41.8, A41.80, A41.88, A41.9, A42.7, B00.7, B37.7, P36.0, P36.1, P36.2, P36.3, P36.4, P36.5, P36.8, P36.9, P35.2, P37.2, P37.5, **A04.7, B95.48, B95.6, B96.2, J18.9, J44.0, N39.0** | **R57.2 septic shock** |
|  | OR |
|  | Any of the codes to the left, plus any of the below: |
|  | *Respiratory*: J96.0, J96.9, J80, R09.2 |
|  | *Cardiovascular*: R57.0, R57.1, R57.8, R57.9, I95.1, I95.8, I95.9 |
|  | *Renal*: N17.0, N17.1, N17.2, N17.8, N17.9 |
|  | *Hepatic*: K72.0, K72.9, K76.3 |
|  | *Neurologic*: F05.0, F05.9, G93.1, G93.4, G93.80 |
|  | *Hematologic*: D69.5, D69.6, D65 |
|  | *Intervention codes (CCI)*: 1.GZ.31.CA-ND, 1.GZ.31.CR-ND, 1.GZ.31.GP-ND |
| **FLAG: SEPSIS** | **FLAG: SEPSIS_SEVERE** |

CIHI = Canadian Institute for Health Information; ICD = International Statistical Classification of Diseases and Related Health Problems, Tenth Revision, Canada

All codes identified as diagnosis type: ‘M, 1, 2, W, X, or Y’.

**Bolded codes** = unique to Jolley sepsis algorithm

b) Description of Codes Used to Define Sepsis Cases

| **Code** | **Description** |
| --- | --- |
| A03.9 | Shigellosis, unspecified |
| A02.1 | Salmonella sepsis |
| A20.7 | Septicaemic plague |
| A21.7 | Generalized tularaemia |
| A22.7 | Anthrax sepsis |
| A23.9 | Brucellosis, unspecified |
| A24.1 | Acute and fulminating melioidosis |
| A26.7 | Erysipelothrix sepsis |
| A28.0 | Pasteurellosis |
| A28.2 | Extraintestinal yersiniosis |
| A32.7 | Listerial sepsis |
| A39.2 | Acute meningococcaemia |
| A39.3 | Chronic meningococcaemia |
| A39.4 | Meningococcaemia, unspecified |
| A40 | Streptococcal sepsis |
| A40.0 | Sepsis due to streptococcus, group A |
| A40.1 | Sepsis due to streptococcus, group B |
| A40.2 | Sepsis due to streptococcus, group D |
| A40.3 | Sepsis due to Streptococcus pneumoniae |
| A04.7 | Enterocolitis due to Clostridium difficile |
| A40.8 | Other streptococcal sepsis |
| A40.9 | Streptococcal sepsis, unspecified |
| A41 | Other sepsis |
| A41.0 | Sepsis due to Staphylococcus aureus |
| A41.1 | Sepsis due to other specified staphylococcus |
| A41.2 | Sepsis due to unspecified staphylococcus |
| A41.3 | Sepsis due to Haemophilus influenzae |
| A41.4 | Sepsis due to anaerobes |
| A41.5 | Sepsis due to other Gram-negative organisms |
| A41.50 | Sepsis due to Escherichia coli [E.coli] |
| A41.51 | Sepsis due to Pseudomonas |
| A41.52 | Sepsis due to Serratia |
| A41.58 | Sepsis due to other gram-negative organisms  Includes: Gram-negative sepsis NOS |
| A41.8 | Other specified sepsis |
| A41.80 | Sepsis due to enterococcus  Excludes: due to Streptococcus D (A40.2) |
| A41.88 | Other specified sepsis |
| A41.9 | Sepsis, unspecified  Includes: Septicaemia |
| A42.7 | Actinomycotic sepsis |
| B00.7 | Disseminated herpesviral disease  Includes: Herpesviral sepsis |
| B37.7 | Candidal sepsis |
| B95.48 | Other streptococcus as the cause of diseases classified to other chapters |
| B95.6 | Staphylococcus aureus as the cause of diseases classified to other chapters |
| B96.2 | Escherichia coli [E. coli] as the cause of diseases classified to other chapters |
| J18.9 | Pneumonia, unspecified |
| J44.0 | Chronic obstructive pulmonary disease with acute lower respiratory infection |
| N39.0 | Urinary tract infection, site not specified |
| P36.0 | Sepsis of newborn due to streptococcus, group B |
| P36.1 | Sepsis of newborn due to other and unspecified streptococci |
| P36.2 | Sepsis of newborn due to Staphylococcus aureus |
| P36.3 | Sepsis of newborn due to other and unspecified staphylococci |
| P36.4 | Sepsis of newborn due to Escherichia coli |
| P36.5 | Sepsis of newborn due to anaerobes |
| P36.8 | Other bacterial sepsis of newborn |
| P36.9 | Bacterial sepsis of newborn, unspecified |
| P35.2 | Congenital herpesviral [herpes simplex] infection |
| P37.2 | Neonatal (disseminated) listeriosis |
| P37.5 | Neonatal candidiasis |
| **Codes Specific for Severe Sepsis** | |
| **Code** | **Description** |
| R57.2 | Septic shock |
| J96.0 | Acute respiratory failure |
| J96.9 | Respiratory failure, unspecified |
| J80 | Adult respiratory distress syndrome |
| R09.2 | Respiratory arrest |
| R57.0 | Cardiogenic shock |
| R57.1 | Hypovolaemic shock |
| R57.2 | Septic shock |
| R57.8 | Other shock |
| R57.9 | Shock, unspecified |
| I95.1 | Orthostatic hypotension |
| I95.8 | Other hypotension |
| I95.9 | Hypotension, unspecified |
| N17.0 | Acute renal failure with tubular necrosis |
| N17.1 | Acute renal failure with acute cortical necrosis |
| N17.2 | Acute renal failure with medullary necrosis |
| N17.8 | Other acute renal failure |
| N17.9 | Acute renal failure, unspecified |
| K72.0 | Acute and subacute hepatic failure |
| K72.9 | Hepatic failure, unspecified |
| K76.3 | Infarction of liver |
| F05.0 | Delirium not superimposed on dementia, so described |
| F05.9 | Other delirium |
| G93.1 | Anoxic brain damage, not elsewhere classified |
| G93.4 | Encephalopathy, unspecified |
| G93.80 | Metabolic encephalopathy |
| D69.5 | Secondary thrombocytopenia |
| D69.6 | Thrombocytopenia, unspecified |
| D65 | Disseminated intravascular coagulation [defibrination syndrome] |
| 1.GZ.31.CA-ND | Ventilation, respiratory system NEC, invasive per orifice approach by endotracheal intubation, positive pressure (e.g. CPAP, BIPAP) |
| 1.GZ.31.CR-ND | Ventilation, respiratory system NEC, invasive per orifice with incision approach for intubation through tracheostomy, positive pressure (e.g. CPAP, BIPAP) |
| 1.GZ.31.GP-ND | Ventilation, respiratory system NEC, Invasive percutaneous  Transluminal approach (e.g. transtracheal jet) through needle, positive pressure (e.g. CPAP, BIPAP) |

**References**

1. Jolley RJ, Quan H, Jetté N, et al. Validation and optimisation of an ICD-10-coded case definition for sepsis using administrative health data. BMJ Open. 2015 Dec 23;5(12):e009487.

# **Supplementary Appendix 3: List of Covariates and Datasets**

| **Covariate** | **Source** |
| --- | --- |
| Age (years) at index | Registered Persons Database (RPDB) |
| Female | RPDB |
| Immigrated prior to index date | Immigration Refugees and Citizenship Canada’s Permanent Resident Database |
| Rural (y/n) | Census |
| Neighbourhood income quintiles | Census |
| ON-MARG: Households and dwellings quintiles | Ontario Marginalization Index |
| ON-MARG: Material resources quintiles | Ontario Marginalization Index |
| ON-MARG: Age and labour force quintiles | Ontario Marginalization Index |
| ON-MARG: Racialized and newcomer populations (Quintile 1 - least deprived) | Ontario Marginalization Index |
| Resource utilization bands | The Johns Hopkins ACG® System Version 10 |
| Frailty indicator (ACGs) | The Johns Hopkins ACG® System Version 10 |
| Number of ACGs (continuous) | The Johns Hopkins ACG® System Version 10 |
| History of cancer diagnosis | Ontario Cancer Registry |
| History of congestive heart failure | ICES Derived Cohort. Reference:  Schultz SE, Rothwell DM, Chen Z, Tu K. Identifying cases of congestive heart failure from administrative data: a validation study using primary care patient records. Chronic diseases and injuries in Canada 2013;33:160-6. |
| History of chronic kidney disease or chronic dialysis | ICES Derived Cohort. Reference:  Fleet JL, Dixon SN, Shariff SZ, Quinn RR, Nash DM, Harel Z, Garg AX. Detecting chronic kidney disease in population-based administrative databases using an algorithm of hospital encounter and physician claim codes. BMC Nephrol. 2013 Apr 5;14:81. |
| History of chronic obstructive pulmonary disease | ICES Derived Cohort. Reference:  Gershon A, Wang C, Guan J, Vasilevska-Ristovska J, Cicutto L, To T. Identifying individuals with physician diagnosed COPD in health administrative databases. COPD 2009;6:388-94. |
| History of diabetes | Ontario Diabetes Dataset |
| No. of ED visits in 1-year prior to index | National Ambulatory Care Reporting System (NACRS) |
| Receipt of or number of home care in 1-year prior to index | HCD |
| Any hospitalization or number of hospitalization episodes in 1-year prior to index | CIHI Discharge Abstract Database (CIHI-DAD) |
| No. of physician visits in 1-year prior to index | Ontario Health Insurance Program Database of Physician Billings |
| Long-term care resident in 2-year lookback | CCRS, ODB, OIHP |
| Hospital size (Teaching) | CIHI-DAD, INST |
| Type of hospitalization (Medical [including mental health]) | CIHI-DAD |
| Charlson score of index hospitalization | CIHI-DAD |
| Hospitalization episode length of stay (days) | CIHI-DAD |
| ICU Admission during index admission | CIHI-DAD |
| Receipt of mechanical ventilation (invasive or non-invasive) | CIHI-DAD |
| Received ECLS during index admission | CIHI-DAD |
| Discharge location (Transferred to acute inpatient facility) | CIHI-DAD |
| Cost of index hospitalization | CIHI-DAD |

**Supplementary Appendix 4: Covariates Included in Propensity Score Model**

| **Variable name** | **Definition** | **Source** | **Scal** | **Unit & Valid range/levels** |
| --- | --- | --- | --- | --- |
| Age | Patient’s age at time of index hospitalization | Registered Persons Database (RPDB) | Continuous | 18–120 Years  Modelled using restricted cubic splines. |
| Sex | Patient’s biological gender | RPDB | Dichotomous | Male or female |
| Immigrant Status | Immigration to Ontario, Canada prior to index date | Immigration Refugees and Citizenship Canada’s Permanent Resident Database | Dichotomous | Immigrant Y/N |
| Long-term care status | Type of setting a patient lives in prior to index hospitalization | CCRS  ODB  OHIP | Dichotomous | Long-term care vs community setting |
| Residence location | Whether patient lives in a rural or urban area based on postal code | RPDB, PCCF (Postal Code Conversion File) | Dichotomous | Rural vs urban |
| Material Resources | Material resources domain of the Ontario Marginalization Index collected at the dissemination area based on postal code | Ontario Marginalization Index | Ordinal quintiles | 1 – least deprived  to  5 – most deprived |
| Aggregated Diagnostic Groups | Weighted score of 32 Johns Hopkins Aggregated Diagnosis Groups® (ADGs) (2 years prior to index admission) | The Johns Hopkins ACG® System Version 10. | Continuous | −37 to 76 |
| Cancer | Inclusion in Ontario Cancer Registry of cancer cases prior to index admission in past 5 years | ICES OCRI | Dichotomous | Cancer (Yes or No) |
| Diabetes | Inclusion in Ontario Diabetes Dataset prior to index admission | ICES ODD | Dichotomous | Diabetes (Yes or No) |
| Chronic Obstructive Pulmonary Disease (COPD) | Inclusion in COPD ICES-derived cohort prior to index admission | ICES COPD | Dichotomous | COPD (Yes or No) |
| Congestive Heart Failure (CHF) | Inclusion in CHF ICES-derived cohort prior to index admission | ICES CHF | Dichotomous | CHF (Yes or No) |
| Chronic Kidney Disease | Codes for chronic kidney disease or dialysis used by Ontario Renal Reporting System in 12 months prior to index admission | Ontario Renal Reporting System | Dichotomous | Chronic kidney disease (Yes or No) |
| Hospitalisation in past year | Hospital admission in the 12 months prior to index admission | CIHI-DAD | Dichotomous | Hospitalization (Yes or No) |
| ER visit in past year | Number of visits made to the emergency room by a patient in the 12 months prior to index admission | NACRS | Continuous | Count: 0 to 365 |
| Physician visits in past year | Number of visits a patient made to physicians in the 12 months prior to index admission | OHIP | Continuous | Count: 0 to 365 |
| Homecare use in past year | Use of homecare services in the 12 months prior to index admission | HCD | Dichotomous | Homecare (Yes or No) |
| Admission to Intensive Care Unit during index admission |  | DAD | Dichotomous | ICU admission (Yes or No) |
| Previously in top 10% of healthcare spending | Ever identified as a HCU using the top 10% cost threshold, in any 30-day interval in the two-year lookback period. | Calculated | Dichotomous | Previous HCU (Yes or No) |

**Supplementary Appendix 5: Classification of High-Cost User**

We identified the cost threshold above which an individual would be among the top 5% of the Ontario population for healthcare spending for that given month. In brief, for each calendar month between January 2014 (earliest date of our lookback period) and December 2019 (end of observation) we identified all individuals who were alive and eligible for OHIP for at least one day and calculated their total healthcare costs. We then identified the individual at the 95th percentile for monthly healthcare costs and used their healthcare costs as the HCU threshold for the top 5%. We also repeated this method for the 50^th^, 90^th^, and 99^th^ percentile. We then calculated the ‘per day’ threshold by dividing the monthly threshold by the number of days in the month.

To identify HCUs within our cohort, we collected the healthcare costs in 30-day intervals starting at the day after discharge from hospital until death, ineligibility for healthcare in Ontario, or administrative censoring on December 31, 2019. We used 30-day intervals to avoid misclassification bias, for example and individual only alive for one month in a year may have had high costs for the month, but total costs may not have met the yearly threshold. In this instance a high-cost user would not have been assigned this status.

We compared the sum of each individual’s 30-day costs against the top 5% HCU threshold for the calendar month corresponding to the last day of the 30-day interval. For intervals with <30 days of observation time, we compared their <30 day interval costs with the product of the ‘per day’ threshold multiplied by the number of observation days in that interval. For example, if an individual was discharged from an acute hospital admission on May 15 2017, their first 30-day interval would be from May 16 2017 to June 15 2017. Total cost for this interval would be compared to HCU threshold for June 2017. If for the subsequent interval the individual was only observed for 17 days (i.e., June 16 to July 3, 2017), the total cost would be compared to the product of the ‘per day’ threshold for July 2017 multiplied by 17. Individuals whose costs were equal to or above the 5% or 1% thresholds were in the top 5% or 1% of the population for total healthcare spending for that period and were classified as a top 5% or top 1% High-Cost User for that observation interval.

We used a lower threshold to identify previous high-cost users (top 10% of the population for total healthcare spending for each 30-day interval period) in the one-year lookback. This decision was based on previous research demonstrating that high-cost user status is often durable, and that previous healthcare utilization predicts future healthcare utilization.

# **Supplementary Appendix 6: Monthly Top 5% and Top 1% High-Cost User Thresholds for 2014-2019 in 2020 Canadian dollars.**

**Top 5%**

| Month | 2014 | 2015 | 2016 | 2017 | 2018 | 2019 |
| --- | --- | --- | --- | --- | --- | --- |
| January | $781 | $774 | $767 | $767 | $799 | $793 |
| February | $704 | $697 | $749 | $701 | $720 | $697 |
| March | $761 | $790 | $788 | $795 | $778 | $767 |
| April | $794 | $797 | $796 | $734 | $775 | $799 |
| May | $814 | $787 | $816 | $835 | $840 | $837 |
| June | $784 | $818 | $814 | $805 | $780 | $759 |
| July | $786 | $772 | $718 | $736 | $763 | $793 |
| August | $695 | $710 | $744 | $744 | $744 | $724 |
| September | $803 | $792 | $772 | $753 | $736 | $766 |
| October | $849 | $812 | $781 | $812 | $840 | $848 |
| November | $771 | $800 | $822 | $817 | $816 | $796 |
| December | $738 | $731 | $720 | $700 | $703 | $731 |

**Top 1%**

| Month | 2014 | 2015 | 2016 | 2017 | 2018 | 2019 |
| --- | --- | --- | --- | --- | --- | --- |
| January | $4495 | $5014 | $4989 | $4993 | $5179 | $5317 |
| February | $4503 | $4529 | $4762 | $4537 | $4689 | $4795 |
| March | $4918 | $5012 | $5053 | $5063 | $5118 | $5233 |
| April | $4913 | $4882 | $4917 | $4809 | $5001 | $5224 |
| May | $5076 | $4988 | $5043 | $5149 | $5257 | $5413 |
| June | $4869 | $4928 | $4955 | $4997 | $5011 | $5139 |
| July | $5001 | $4994 | $4891 | $4965 | $5187 | $5363 |
| August | $4859 | $4861 | $4973 | $5019 | $5191 | $5248 |
| September | $4932 | $4932 | $4923 | $4916 | $5037 | $5199 |
| October | $5145 | $5045 | $5025 | $5127 | $5391 | $5505 |
| November | $4867 | $4918 | $5014 | $5059 | $5244 | $5297 |
| December | $4967 | $4928 | $4934 | $4929 | $5113 | $5279 |

# **Supplementary Appendix 7: Baseline characteristics stratified by previous healthcare costs (top 10%) pre and post propensity score weighting**

**7.a Baseline characteristics before and after IPTW weighting for individuals who were previously in top 10% of healthcare spending.**

|  | **Before Weighting** | | | **After Weighting** | | |
| --- | --- | --- | --- | --- | --- | --- |
| **Variable** | **Sepsis**  **N = 75,105** | **Non-Sepsis** **N = 782,399** | **Standardized Difference** | **Sepsis**  **N=821,753** | **Non-Sepsis** **N=858,653** | **Weighted Standardized Difference** |
| **Age,** mean ± SD  median (IQR) | 73.24 ± 16.00  76 (65-85) | 61.03 ± 18.89  64 (48-75) | 0.7  0.71 | 62.97 | 62.13 | 0.044 |
| **Female,** n (%) | 42,542 (56.6%) | 428,948 (54.8%) | 0.04 | 52.74% | 54.98% | 0.045 |
| **Immigration Status,** n (%) | | | | | | |
| Immigrated prior to index date | 6,106 (8.1%) | 95,185 (12.2%) | 0.13 | 10.93% | 11.80% | 0.027 |
| Non-immigrant | 68,999 (91.9%) | 687,214 (87.8%) | 0.13 | 89.07% | 88.20% | 0.027 |
| Recent immigrant (≤ 5 years) | 351 (0.5%) | 9,563 (1.2%) | 0.08 | 0.79% | 1.16% | 0.038 |
| Long-term immigrant (>5 years) | 5,755 (7.7%) | 85,622 (10.9%) | 0.11 | 10.14% | 10.64% | 0.016 |
| **Rural,** n (%) | 7,364 (9.8%) | 75,735 (9.7%) | 0 | 9.83% | 9.69% | 0.005 |
| **Income Quintile^a^, n (%)** | | | | | | |
| 1 (lowest) | 19,801 (26.4%) | 174,502 (22.3%) | 0.09 | 24.27% | 22.68% | 0.037 |
| 2 | 16,509 (22.0%) | 163,903 (20.9%) | 0.03 | 21.46% | 21.03% | 0.010 |
| 3 | 14,256 (19.0%) | 154,123 (19.7%) | 0.02 | 19.46% | 19.62% | 0.004 |
| 4 | 12,560 (16.7%) | 144,670 (18.5%) | 0.05 | 17.74% | 18.32% | 0.015 |
| 5 (highest) | 11,627 (15.5%) | 142,699 (18.2%) | 0.07 | 16.71% | 18.01% | 0.035 |
| Missing information | 352 (0.5%) | 2,502 (0.3%) | 0.02 | 0.37% | 0.34% | 0.004 |
| **ONTARIO MARGINALIZATION INDEX DIMENSIONS** | | | | | | |
| **Household and Dwellings, n (%)** | | | | | | |
| 1 (least deprived) | 9,602 (12.8%) | 131,261 (16.8%) | 0.11 | 15.23% | 16.43% | 0.033 |
| 2 | 12,079 (16.1%) | 140,561 (18.0%) | 0.05 | 17.22% | 17.78% | 0.015 |
| 3 | 13,803 (18.4%) | 148,375 (19.0%) | 0.02 | 18.44% | 18.91% | 0.012 |
| 4 | 16,080 (21.4%) | 156,491 (20.0%) | 0.03 | 20.40% | 20.13% | 0.007 |
| 5 (most deprived) | 22,393 (29.8%) | 196,549 (25.1%) | 0.11 | 27.35% | 25.54% | 0.041 |
| Missing information | 1,148 (1.5%) | 9,162 (1.2%) | 0.03 | 1.35% | 1.21% | 0.013 |
| **Material Resources,n (%)** | | | | | | |
| 1 (least deprived) | 12,818 (17.1%) | 153,006 (19.6%) | 0.06 | 18.32% | 19.32% | 0.026 |
| 2 | 13,748 (18.3%) | 152,377 (19.5%) | 0.03 | 18.72% | 19.35% | 0.016 |
| 3 | 14,106 (18.8%) | 149,574 (19.1%) | 0.01 | 19.03% | 19.09% | 0.002 |
| 4 | 15,249 (20.3%) | 154,240 (19.7%) | 0.01 | 20.14% | 19.77% | 0.009 |
| 5 (most deprived) | 18,036 (24.0%) | 164,040 (21.0%) | 0.07 | 22.43% | 21.25% | 0.029 |
| Missing information | 1,148 (1.5%) | 9,162 (1.2%) | 0.03 | 1.35% | 1.21% | 0.013 |
| **Age and Labour Force, n (%)** |  |  |  |  |  |  |
| 1 (least deprived) | 10,210 (13.6%) | 147,418 (18.8%) | 0.14 | 17.31% | 18.39% | 0.028 |
| 2 | 11,700 (15.6%) | 140,496 (18.0%) | 0.06 | 17.53% | 17.76% | 0.006 |
| 3 | 12,482 (16.6%) | 137,158 (17.5%) | 0.02 | 17.57% | 17.43% | 0.004 |
| 4 | 14,570 (19.4%) | 145,833 (18.6%) | 0.02 | 18.92% | 18.67% | 0.006 |
| 5 (most deprived) | 24,995 (33.3%) | 202,332 (25.9%) | 0.16 | 27.31% | 26.53% | 0.017 |
| Missing information | 1,148 (1.5%) | 9,162 (1.2%) | 0.03 | 1.35% | 1.21% | 0.013 |
| **Racialized and Newcomer Populations, n (%)** | | | | | | |
| 1 (least deprived) | 17,213 (22.9%) | 163,947 (21.0%) | 0.05 | 21.38% | 21.12% | 0.006 |
| 2 | 15,799 (21.0%) | 151,782 (19.4%) | 0.04 | 20.19% | 19.49% | 0.018 |
| 3 | 13,733 (18.3%) | 143,659 (18.4%) | 0 | 18.14% | 18.36% | 0.006 |
| 4 | 12,932 (17.2%) | 146,943 (18.8%) | 0.04 | 17.84% | 18.69% | 0.022 |
| 5 (most deprived) | 14,280 (19.0%) | 166,906 (21.3%) | 0.06 | 21.09% | 21.13% | 0.001 |
| Missing information | 1,148 (1.5%) | 9,162 (1.2%) | 0.03 | 1.35% | 1.21% | 0.013 |
| **Resource Utilization, n (%)** | | | | | | |
| Band 0 – Non Users | 129 (0.2%) | 396 (0.1%) | 0.04 | 0.13% | 0.05% | 0.026 |
| Band 1 | 200 (0.3%) | 1,480 (0.2%) | 0.02 | 0.40% | 0.18% | 0.040 |
| Band 2 | 1,469 (2.0%) | 19,502 (2.5%) | 0.04 | 2.77% | 2.42% | 0.022 |
| Band 3 | 23,825 (31.7%) | 296,672 (37.9%) | 0.13 | 36.35% | 37.34% | 0.020 |
| Band 4 | 22,184 (29.5%) | 275,902 (35.3%) | 0.12 | 30.49% | 34.90% | 0.094 |
| Band 5 – Very High Users | 27,298 (36.3%) | 188,447 (24.1%) | 0.27 | 29.87% | 25.11% | 0.107 |
| **Frailty indicator** (ACGs), n (%) | 17,543 (23.4%) | 69,145 (8.8%) | 0.4 | 13.81% | 9.91% | 0.121 |
| **Number of ACGs**, mean ± SD,  median (IQR) | 8.99 ± 3.86  9 (6-12) | 8.41 ± 3.60  8 (6-11) | 0.16  0.16 | 8.72 | 8.47 | 0.068 |
| **Number of ACGs** (0), n (%) | 129 (0.2%) | 410 (0.1%) | 0.04 | 0.13% | 0.06% | 0.025 |
| **Number of ACGs** (1-9), n (%) | 42,171 (56.1%) | 498,595 (63.7%) | 0.16 | 59.34% | 63.05% | 0.076 |
| **Number of ACGs** (10-19), n (%) | 32,544 (43.3%) | 281,665 (36.0%) | 0.15 | 40.24% | 36.63% | 0.074 |
| **Number of ACGs** (20-29), n (%) | 261 (0.3%) | 1,729 (0.2%) | 0.02 | 0.28% | 0.27% | 0.003 |
| **Prior Cancer**, n (%), | 17,996 (24.0%) | 150,741 (19.3%) | 0.11 | 20.58% | 19.69% | 0.022 |
| **Prior Congestive Heart Failure**, n (%) | 14,559 (19.4%) | 73,580 (9.4%) | 0.29 | 11.51% | 10.34% | 0.038 |
| **Prior chronic kidney disease or chronic dialysis**, n (%) | 8,547 (11.4%) | 47,422 (6.1%) | 0.19 | 7.74% | 6.56% | 0.046 |
| **Prior chronic obstructive pulmonary disease,** n (%) | 26,281 (35.0%) | 154,215 (19.7%) | 0.35 | 23.13% | 21.11% | 0.049 |
| **Prior diabetes,** n (%) | 27,733 (36.9%) | 197,419 (25.2%) | 0.25 | 28.10% | 26.31% | 0.040 |
| **Healthcare Use 1-year Prior Index** | | | | | | |
| ED visits, mean ± SD,   median (IQR) | 1.65 ± 2.55  1 (0-2) | 1.36 ± 2.68  1 (0-2) | 0.11  0.24 | 1.64 | 1.49 | 0.033 |
| Receipt of home care, n (%) | 27,135 (36.1%) | 131,786 (16.8%) | 0.45 | 21.17% | 18.64% | 0.063 |
| Hospitalization, n (%) | 6,983 (9.3%) | 53,320 (6.8%) | 0.09 | 8.06% | 7.07% | 0.037 |
| No. of hospitalization episodes, mean ± SD, median (IQR) | 0.14 ± 0.51  0 (0-0) | 0.10 ± 0.46  0 (0-0) | 0.07  0.09 | 0.12 | 0.11 | 0.026 |
| No. of physician visits, mean ± SD, median (IQR) | 11.99 ± 10.65  10 (5-16) | 12.01 ± 9.57  10 (6-16) | 0  0.04 | 12.8 | 12.02 | 0.065 |
| **LTC resident in 2-year lookback**, n (%) | 6,438-6,442 (8.6%) | 15,218 (1.9%) | 0.3 | 3.09% | 2.59% | 0.030 |

*SD* Standard Deviation; *IQR* Interquartile Range; *ACG Adjusted Clinical Groups, Frailty, and Resource utilization bands from* the Johns Hopkins ACG® System Version 10; OnMARG Ontario Marginalization Index; LTC Long-term Care Resident.

**7b. Baseline characteristics before and after IPTW weighting for individuals who were not previously in the top 10% for healthcare costs.**

|  | **Before Weighting** | | | **After Weighting** | | |
| --- | --- | --- | --- | --- | --- | --- |
| **Variable** | **Sepsis**  **N = 3,960** | **Non-Sepsis** **N = 65,593** | **Standardized Difference** | **Sepsis** **N = 66,961** | **Non-Sepsis** **N = 69,578** | **Weighted Standardized Difference** |
| **Age,** mean ± SD  median (IQR) | 63.94 ± 18.32  64 (53-78) | 50.23 ± 18.29  51 (35-63) | 0.75  0.74 | 52.08 | 51.03 | 0.056 |
| **Female,** n (%) | 2,014 (50.9%) | 28,984 (44.2%) | 0.13 | 40.26% | 44.57% | 0.087 |
| **Immigration Status,** n (%) | | | | | | |
| Immigrated prior to index date | 390 (9.8%) | 9,520 (14.5%) | 0.14 | 14.40% | 14.25% | 0.004 |
| Non-immigrant | 3,570 (90.2%) | 56,073 (85.5%) | 0.14 | 85.60% | 85.75% | 0.004 |
| Recent immigrant (≤ 5 years) | 36 (0.9%) | 1,138 (1.7%) | 0.07 | 1.47% | 1.69% | 0.017 |
| Long-term immigrant (>5 years) | 354 (8.9%) | 8,382 (12.8%) | 0.12 | 12.93% | 12.56% | 0.011 |
| **Rural,** n (%) | 470 (11.9%) | 6,353 (9.7%) | 0.07 | 9.92% | 9.82% | 0.003 |
| **Income Quintile^a^, n (%)** | | | | | | |
| 1 (lowest) | 997 (25.2%) | 13,025 (19.9%) | 0.13 | 21.65% | 20.14% | 0.037 |
| 2 | 930 (23.5%) | 13,442 (20.5%) | 0.07 | 21.63% | 20.61% | 0.025 |
| 3 | 737 (18.6%) | 13,281 (20.2%) | 0.04 | 19.45% | 20.18% | 0.018 |
| 4 | 702 (17.7%) | 13,008 (19.8%) | 0.05 | 20.65% | 19.68% | 0.024 |
| 5 (highest) | 573 (14.5%) | 12,586 (19.2%) | 0.13 | 16.21% | 18.99% | 0.073 |
| Missing information | 21 (0.5%) | 251 (0.4%) | 0.02 | 0.42% | 0.40% | 0.004 |
| **ONTARIO MARGINALIZATION INDEX DIMENSIONS** | | | | | | |
| **Household and Dwellings, n (%)** | | | | | | |
| 1 (least deprived) | 568 (14.3%) | 12,602 (19.2%) | 0.13 | 17.88% | 18.94% | 0.027 |
| 2 | 674 (17.0%) | 12,684 (19.3%) | 0.06 | 19.11% | 19.22% | 0.003 |
| 3 | 762 (19.2%) | 12,912 (19.7%) | 0.01 | 18.43% | 19.68% | 0.032 |
| 4 | 832 (21.0%) | 12,602 (19.2%) | 0.04 | 19.40% | 19.36% | 0.001 |
| 5 (most deprived) | 1,041 (26.3%) | 13,858 (21.1%) | 0.12 | 23.62% | 21.34% | 0.055 |
| Missing information | 83 (2.1%) | 935 (1.4%) | 0.05 | 1.56% | 1.46% | 0.008 |
| **Material Resources,n (%)** | | | | | | |
| 1 (least deprived) | 616 (15.6%) | 13,233 (20.2%) | 0.12 | 18.67% | 19.90% | 0.031 |
| 2 | 709 (17.9%) | 13,229 (20.2%) | 0.06 | 19.55% | 20.03% | 0.012 |
| 3 | 723 (18.3%) | 12,785 (19.5%) | 0.03 | 20.14% | 19.43% | 0.018 |
| 4 | 877 (22.1%) | 12,721 (19.4%) | 0.07 | 19.61% | 19.55% | 0.002 |
| 5 (most deprived) | 952 (24.0%) | 12,690 (19.3%) | 0.11 | 20.46% | 19.62% | 0.021 |
| Missing information | 83 (2.1%) | 935 (1.4%) | 0.05 | 1.56% | 1.46% | 0.008 |
| **Age and Labour Force, n (%)** |  |  |  |  |  |  |
| 1 (least deprived) | 678 (17.1%) | 14,839 (22.6%) | 0.14 | 23.00% | 22.26% | 0.018 |
| 2 | 713 (18.0%) | 12,911 (19.7%) | 0.04 | 20.01% | 19.58% | 0.011 |
| 3 | 708 (17.9%) | 12,100 (18.4%) | 0.01 | 18.01% | 18.43% | 0.011 |
| 4 | 754 (19.0%) | 11,803 (18.0%) | 0.03 | 17.51% | 18.07% | 0.014 |
| 5 (most deprived) | 1,024 (25.9%) | 13,005 (19.8%) | 0.14 | 19.89% | 20.20% | 0.008 |
| Missing information | 83 (2.1%) | 935 (1.4%) | 0.05 | 1.56% | 1.46% | 0.008 |
| **Racialized and Newcomer Populations, n (%)** | | | | | | |
| 1 (least deprived) | 953 (24.1%) | 13,283 (20.3%) | 0.09 | 19.43% | 20.49% | 0.027 |
| 2 | 847 (21.4%) | 12,765 (19.5%) | 0.05 | 20.20% | 19.54% | 0.016 |
| 3 | 709 (17.9%) | 11,992 (18.3%) | 0.01 | 17.26% | 18.28% | 0.027 |
| 4 | 612 (15.5%) | 12,360 (18.8%) | 0.09 | 17.06% | 18.71% | 0.043 |
| 5 (most deprived) | 756 (19.1%) | 14,258 (21.7%) | 0.07 | 24.49% | 21.52% | 0.071 |
| Missing information | 83 (2.1%) | 935 (1.4%) | 0.05 | 1.56% | 1.46% | 0.008 |
| **Resource Utilization, n (%)** | | | | | | |
| Band 0 – Non Users | 797 (20.1%) | 9,052 (13.8%) | 0.17 | 15.18% | 14.06% | 0.032 |
| Band 1 | 257 (6.5%) | 4,056 (6.2%) | 0.01 | 7.60% | 6.16% | 0.057 |
| Band 2 | 845 (21.3%) | 14,597 (22.3%) | 0.02 | 21.55% | 22.29% | 0.018 |
| Band 3 | 1,906 (48.1%) | 30,533 (46.5%) | 0.03 | 50.38% | 46.59% | 0.076 |
| Band 4 | 132 (3.3%) | 7,164 (10.9%) | 0.3 | 4.21% | 10.61% | 0.246 |
| Band 5 – Very High Users | 23 (0.6%) | 191 (0.3%) | 0.04 | 1.08% | 0.29% | 0.095 |
| **Frailty indicator** (ACGs), n (%) | 128 (3.2%) | 485 (0.7%) | 0.18 | 1.41% | 0.82% | 0.056 |
| **Number of ACGs**, mean ± SD,  median (IQR) | 2.64 ± 2.27  2 (1-4) | 3.11 ± 2.32  3 (1-5) | 0.2  0.21 | 3.2 | 3.08 | 0.047 |
| **Number of ACGs** (0), n (%) | 799 (20.2%) | 9,060 (13.8%) | 0.17 | 15.21% | 14.07% | 0.032 |
| **Number of ACGs** (1-9), n (%) | 3,131 (79.1%) | 55,899 (85.2%) | 0.16 | 82.72% | 84.99% | 0.062 |
| **Number of ACGs** (10-19), n (%) | 30 (0.8%) | 634 (1.0%) | 0.02 | 2.06% | 0.94% | 0.093 |
| **Number of ACGs** (20-29), n (%) | 0 | 0 | n/a | n/a | n/a | n/a |
| **Prior Cancer**, n (%), | 264 (6.7%) | 2,321 (3.5%) | 0.14 | 4.20% | 3.73% | 0.024 |
| **Prior Congestive Heart Failure**, n (%) | 152 (3.8%) | 803 (1.2%) | 0.17 | 1.60% | 1.38% | 0.018 |
| **Prior chronic kidney disease or chronic dialysis**, n (%) | 24 (0.6%) | 182 (0.3%) | 0.05 | 0.47% | 0.30% | 0.028 |
| **Prior chronic obstructive pulmonary disease,** n (%) | 682 (17.2%) | 4,816 (7.3%) | 0.3 | 8.97% | 7.92% | 0.038 |
| **Prior diabetes,** n (%) | 641 (16.2%) | 5,817 (8.9%) | 0.22 | 10.84% | 9.31% | 0.051 |
| **Healthcare Use 1-year Prior Index** | | | | | | |
| ED visits, mean ± SD,   median (IQR) | 0.07 ± 0.28  0 (0-0) | 0.10 ± 0.35  0 (0-0) | 0.09  0.08 | 0.1 | 0.1 | 0.004 |
| Receipt of home care, n (%) | 125 (3.2%) | 536 (0.8%) | 0.17 | 1.03% | 0.96% | 0.008 |
| Hospitalization, n (%) | 0 | 0 | n/a | n/a | n/a | n/a |
| No. of hospitalization episodes, mean ± SD, median (IQR) | 0.00 ± 0.00  0 (0-0) | 0.00 ± 0.00  0 (0-0) | n/a | n/a | n/a | n/a |
| No. of physician visits, mean ± SD, median (IQR) | 2.61 ± 3.36  2 (0-4) | 3.59 ± 4.22  2 (0-5) | 0.26  0.24 | 3.39 | 3.54 | 0.036 |
| **LTC resident in 2-year lookback**, n (%) | <=5 (0.1%) | 9 (0.0%) | 0.03 | 0.02% | 0.02% | 0.000 |

*SD* Standard Deviation; *IQR* Interquartile Range; *ACG Adjusted Clinical Groups, Frailty, and Resource utilization bands from* the Johns Hopkins ACG® System Version 10; OnMARG Ontario Marginalization Index; LTC Long-term Care Resident.

Supplementary Appendix 8: The RECORD statement – checklist of items, extended from the STROBE statement, that should be reported in observational studies using routinely collected health data.

|  | **Item No.** | **STROBE items** | **Location in manuscript where items are reported** | **RECORD items** | **Location in manuscript where items are reported** |
| --- | --- | --- | --- | --- | --- |
| **Title and abstract** | | | | | |
|  | 1 | (a) Indicate the study’s design with a commonly used term in the title or the abstract (b) Provide in the abstract an informative and balanced summary of what was done and what was found | Pg. (a) 1, (b) 4 | RECORD 1.1: The type of data used should be specified in the title or abstract. When possible, the name of the databases used should be included.  RECORD 1.2: If applicable, the geographic region and timeframe within which the study took place should be reported in the title or abstract.  RECORD 1.3: If linkage between databases was conducted for the study, this should be clearly stated in the title or abstract. | Pg. 4  Pg. 4  Pg. 4 |
| **Introduction** | | | | | |
| Background rationale | 2 | Explain the scientific background and rationale for the  investigation being reported | Pg. 6 |  |  |
| Objectives | 3 | State specific objectives, including any prespecified hypotheses | Pg. 6 |  |  |
| **Methods** | | | | | |
| Study Design | 4 | Present key elements of study design early in the paper | Pg. 7 |  |  |
| Setting | 5 | Describe the setting, locations, and relevant dates, including  periods of recruitment, exposure, follow-up, and data collection | Pg. 7-9 |  |  |

| Participants | 6 | 1. *Cohort study* - Give the eligibility criteria, and the sources and methods of selection of participants. Describe methods of follow-up   *Case-control study* - Give the eligibility criteria, and the sources and methods of case ascertainment and control selection. Give the rationale for the choice of cases and controls *Cross-sectional study* - Give the eligibility criteria, and the sources and methods of selection of participants   1. *Cohort study* - For matched studies, give matching criteria and number of exposed and unexposed   *Case-control study* - For matched studies, give matching criteria and the number of controls per case |  | RECORD 6.1: The methods of study population selection (such as codes or algorithms used to identify subjects) should be listed in detail. If this is not possible, an explanation should be provided.  RECORD 6.2: Any validation studies of the codes or algorithms used to select the population should be referenced. If validation was conducted for this study and not published elsewhere, detailed methods and results should be provided.  RECORD 6.3: If the study involved linkage of databases, consider use of a flow diagram or other graphical display to demonstrate the data linkage process, including the number of individuals with linked data at each stage. | Pg. 7 (exact codes available in supplementary file)  Validated algorithm referenced and ICD-10 codes are available in the supplementary file  Linkage described on pg. 7 |
| --- | --- | --- | --- | --- | --- |
| Variables | 7 | Clearly define all outcomes, exposures, predictors, potential confounders, and effect modifiers. Give diagnostic criteria, if applicable. | Pg. 9 and 10 | RECORD 7.1: A complete list of codes and algorithms used to classify exposures, outcomes, confounders, and effect modifiers should be provided. If these cannot be reported, an explanation should be provided. | Supplementary appendix |
| Data sources/ measurement | 8 | For each variable of interest, give sources of data and details of methods of assessment (measurement).  Describe comparability of assessment methods if there is more than one group | Pg. 7 and 8  Sources of data available in supplementary file |  |  |

| Bias | 9 | Describe any efforts to address potential sources of bias | Pg. 9 |  |  |
| --- | --- | --- | --- | --- | --- |
| Study size | 10 | Explain how the study size was arrived at | Pg. 7 (cohort creation) |  |  |
| Quantitative variables | 11 | Explain how quantitative variables were handled in the analyses. If applicable, describe  which groupings were chosen, and why | Pg. 9 and 10 |  |  |
| Statistical methods | 12 | 1. Describe all statistical methods, including those used to control for confounding 2. Describe any methods used to examine subgroups and interactions 3. Explain how missing data were addressed 4. *Cohort study* - If applicable, explain how loss to follow-up was addressed   *Case-control study* - If applicable, explain how matching of cases and controls was addressed  *Cross-sectional study* - If applicable, describe analytical methods taking account of sampling strategy   1. Describe any sensitivity analyses | Pg. 10 |  |  |
| Data access and cleaning methods |  | .. |  | RECORD 12.1: Authors should describe the extent to which the investigators had access to the database population used to create the study population. | Pg. 7 |

|  |  |  |  | RECORD 12.2: Authors should provide information on the data cleaning methods used in the study. | Pg. 7 |
| --- | --- | --- | --- | --- | --- |
| Linkage |  | .. |  | RECORD 12.3: State whether the study included person-level, institutional-level, or other data linkage across two or more databases. The methods of linkage and methods of linkage quality evaluation should be provided. | Pg. 7 |
| **Results** | | | | | |
| Participants | 13 | 1. Report the numbers of individuals at each stage of the study (*e.g.*, numbers potentially eligible, examined for eligibility, confirmed eligible, included in the study, completing follow-up, and analysed) 2. Give reasons for non- participation at each stage. 3. Consider use of a flow diagram | Pg. 11  Figure 1. Cohort Creation Flow diagram | RECORD 13.1: Describe in detail the selection of the persons included in the study (*i.e.,* study population selection) including filtering based on data quality, data availability and linkage. The selection of included persons can be described in the text and/or by means of the study flow diagram. | Pg. 11 and Figure 1 |
| Descriptive data | 14 | 1. Give characteristics of study participants (*e.g.*, demographic, clinical, social) and information on exposures and potential confounders 2. Indicate the number of participants with missing data for each variable of interest 3. *Cohort study* - summarise follow-up time (*e.g.*, average and total amount) | Pg. 11 |  |  |
| Outcome data | 15 | *Cohort study* - Report numbers of outcome events or summary measures over time  *Case-control study* - Report numbers in each exposure | Pg. 11 |  |  |

|  |  | category, or summary measures of exposure  *Cross-sectional study* - Report numbers of outcome events or summary measures |  |  |  |
| --- | --- | --- | --- | --- | --- |
| Main results | 16 | 1. Give unadjusted estimates and, if applicable, confounder- adjusted estimates and their precision (e.g., 95% confidence interval). Make clear which confounders were adjusted for and why they were included 2. Report category boundaries when continuous variables were categorized 3. If relevant, consider translating estimates of relative risk into absolute risk for a meaningful time period | Pg. 11 and 12 |  |  |
| Other analyses | 17 | Report other analyses done— e.g., analyses of subgroups and interactions, and sensitivity analyses | Pg. 12 |  |  |
| **Discussion** | | | | | |
| Key results | 18 | Summarise key results with  reference to study objectives | Pg. 13 |  |  |
| Limitations | 19 | Discuss limitations of the study, taking into account sources of potential bias or imprecision.  Discuss both direction and magnitude of any potential bias | Pg. 14 | RECORD 19.1: Discuss the implications of using data that were not created or collected to answer the specific research question(s). Include discussion of misclassification bias, unmeasured confounding, missing data, and changing eligibility over time, as they pertain to the study being  reported. | Pg. 14     Supplementary appendix 5  Pg. 7 |
| Interpretation | 20 | Give a cautious overall interpretation of results considering objectives, |  |  |  |

|  |  | limitations, multiplicity of analyses, results from similar studies, and other relevant evidence | Pg. 13 and 14 |  |  |
| --- | --- | --- | --- | --- | --- |
| Generalisability | 21 | Discuss the generalisability (external validity) of the study results | Pg. 14 |  |  |
| **Other Information** | | | | | |
| Funding | 22 | Give the source of funding and the role of the funders for the present study and, if applicable, for the original study on which the present article is based | Pg. 2 |  |  |
| Accessibility of protocol, raw data, and programming  code |  | .. |  | RECORD 22.1: Authors should provide information on how to access any supplemental information such as the study protocol, raw data, or  programming code. | Pg. 2  Pg. 7 |

*Reference: Benchimol EI, Smeeth L, Guttmann A, Harron K, Moher D, Petersen I, Sørensen HT, von Elm E, Langan SM, the RECORD Working Committee. The REporting of studies Conducted using Observational Routinely-collected health Data (RECORD) Statement. *PLoS Medicine* 2015; in press.

*Checklist is protected under Creative Commons Attribution ([CC BY](http://creativecommons.org/licenses/by/4.0/)) license.
